# Baseline 2D PC-MRI hemodynamic markers correlate to aorta growth in serially monitored bicuspid aortic valve patients

**DOI:** 10.1186/1532-429X-16-S1-P90

**Published:** 2014-01-16

**Authors:** Nicholas Naro, Alexander P Taylor, Jyothy Puthumana, James C Carr, Patrick M McCarthy, Michael Markl, Jeremy D Collins, Alex J Barker

**Affiliations:** 1 Feinberg School of Medicine, Chicago, Illinois, USA; 2 Radiology, Feinberg School of Medicine, Chicago, Illinois, USA; 3 Cardiology, Feinberg School of Medicine, Chicago, Illinois, USA; 4 Cardiac Surgery, Feinberg School of Medicine, Chicago, Illinois, USA; 5 McCormick School of Engineering, Evanston, Illinois, USA

## Background

Bicuspid aortic valve (BAV) is the most common congenital heart defect whereby a significant percentage of patients experience dissection or progressive aortic dilation. There is increasing evidence that aortic hemodynamics such as wall shear stress (WSS) and flow jet eccentricity may exacerbate the development of aortopathy; however, these relationships have been investigated using experimental cardiac MRI protocols. We hypothesize that standard 2D through-plane encoded PC-MRI can aid in identifying BAV patients most at-risk for progressive aortic dilation.

## Methods

41 consecutive BAV patients who received routine cardiac MRI surveillance at our institution (147 exams) were enrolled in this retrospective longitudinal study. Enrollment criteria included: the presence of a baseline 2D through-plane encoded PC-MRI exam in the ascending aorta, a contrast-enhanced thoracic MRA (CE-MRA), at least 2 CE-MRA studies dated more than 6 months apart, and age over 18 years. Aortic diameters were measured at the sinuses of valsalva (SOV) and the mid ascending aorta (MAA) on a dedicated workstation using doubly-oblique multiplanar reformatted images. Growth rates were computed at each measurement position. Cohorts were created based the baseline 2D PC-MRI slice position: either at the level of the right pulmonary artery (MAA level) or immediately superior to the SOV. Linear regression analysis was performed to examine the correlation of aortic growth rate with hemodynamic and geometric parameters, including baseline diameter, vessel distension, regurgitant fraction, peak velocity, effective orifice area (EOA), WSS, and flow displacement. A Pearson's correlation coefficient (r) and null hypothesis probability (p) were calculated.

## Results

The average age at baseline was 45.4 ± 9.9 years. The average follow-up was 3.0 ± 1.4 years over 3.7 ± 1.6 scans with an annual growth of 0.3 ± 1.1 mm/yr. A total of 25 baseline exams existed at the MAA and 18 baseline exams at the SOV (2 subjects had both MAA and SOV baseline exams). In the MAA cohort, MAA growth was correlated to EOA (r = 0.51, p = 0.009); SOV growth was correlated to systolic WSS eccentricity (r = 0.46, p = 0.02) and baseline diameter (r = 0.45, p = 0.03). In the SOV cohort, SOV growth was correlated to oscillatory shear index (r = 0.57, p = 0.01); MAA growth was correlated to EOA (r = 0.47, p < 0.05).

## Conclusions

Average annual growth rates were below the resolution limits of CE-MRA; however, growth rates obtained from multiple serial diameter measurements were significantly correlated with hemodynamic metrics. Despite the need for large cohort studies to conclusively demonstrate the relation of aortic growth rate and flow abnormalities, this study shows promising initial results from standard cardiac MR protocols.

## Funding

AHA 13SDG14360004.

